# The Association Between Sexual Harassment and Mental Health Among Chinese College Students: Do Gender and Social Support Matter?

**DOI:** 10.3389/ijph.2022.1604922

**Published:** 2022-09-01

**Authors:** Sasa Wang, Lisa Eklund, Xueyan Yang

**Affiliations:** ^1^ Department of Sociology, Shaanxi Normal University, Xi’an, China; ^2^ Department of Sociology, Lund University, Lund, Sweden; ^3^ The Institute for Population and Development Studies, Xi’an Jiaotong University, Xi’an, China

**Keywords:** mental health, China, higher education, gender, social support, sexual harassment

## Abstract

**Objectives:** This study examined the association between sexual harassment (SH) and college students’ mental health in the Chinese context and its gender differences, exploring the moderating role of social support.

**Methods:** Data were from the Third Survey of Chinese Women’s Social Status and included 5,032 college students. We employed the ordinary least squares (OLS) regression models with interaction terms to report the moderating effects of gender and social support on the association between SH and mental health.

**Results:** Gender harassment and unwelcome sexual attention were negatively associated with mental health among all students, with no observed gender difference. Financial and large-scale emotional support moderated the association between unwelcome sexual attention and women’s mental health but were not buffer factors for men. Learning support aggravated the adverse association between gender harassment and men’s mental health.

**Conclusion:** SH is a significant trigger for men’s and women’s mental health problems. When they are subjected to SH, financial and emotional support are protective resources for women, but learning support is risky for men.

## Introduction

Unwelcome sexual behaviour, both implicit and explicit, is considered sexual harassment (SH), and young people are particularly exposed to it [1, 2]. Between January and May 2022, the Chinese media reported more than 12 cases of SH committed by teachers against students. Social scientists consider SH a chronic stressor detrimental to one’s health and well-being [3–5]. Victims of SH are prone to psychological harm, including anxiety, depression, panic, fear, diminished self-confidence, and low life satisfaction [3], with long-lasting effects [4, 5].

SH may damage the psychological health of Chinese college students more due to society’s conservative sexual culture and greater power disparity in universities compared to Western countries [6]. When being harassed, they may remain silent rather than seek support [6, 7]. Upon disclosing SH experiences, victims are often treated unequally, censured by superiors, or become the target of rumours [6]. SH occasionally appears alongside suicide in Chinese news. However, there are few studies on the health effects of SH in the Chinese context.

The stress exposure argument suggests that women deem stressors more threatening than men [8–10]. Theoretically, this indicates that SH harms the mental health of women more than that of men. However, regarding how responses to SH are gendered, conclusions drawn for different populations in Western countries vary [5, 11–17]. Many Chinese still believe that SH means the harassment of women by men because men are presumably more powerful [1, 18]. Therefore, Chinese men’s mental health is often ignored when they become victims [18]. However, a large percentage of them have experienced SH [7]. They may also be prone to psychological harm because the male-dominated culture makes them particularly reluctant to disclose their experiences and seek help.

The transactional theory of stress and coping (TTSC) proposed by Lazarus and Folkman is a leading theoretical approach to psychological stress and coping research across multiple fields [8, 19, 20]. According to TTSC, stress is a process that involves continuous transactions (or interactions) between a person and their environment. Different individuals respond to the same event with varying intensity or duration, depending on whether their cognitive judgment of the situation is benign or stressful and their ability to manage stress. When the event is appraised as a threatening situation that can potentially lead to harm or loss, coping is needed to address it [8, 19, 20]. Social support has emerged as a crucial coping resource in the literature on traumatic stress because of its relationship with stress outcomes [19, 21–23]. This suggests that social support may reduce individuals’ risk of developing mental health problems when exposed to SH.

### The Relationship Between Sexual Harassment and Mental Health: Gender Differences

TTSC suggests that across varying situations, people express different patterns of stress responses [8, 24]. Hence, the extent to which SH affects health may vary depending on its form. Students may consider contact behaviours more stressful and develop more health problems [12]. Based on severity, Gelfand et al. classified SH into three non-overlapping behavioural dimensions: gender harassment, unwelcome sexual attention, and sexual coercion [25]. This study examines the former two because of their high prevalence [7]. Gender harassment includes lecherous expressions or obscene expressions, discriminatory comments, and inappropriate displays of material, which would offend a particular gender. Unwelcome sexual attention involves unwanted physical contact, aggressive messages or phone calls, non-reciprocal dating requests, and sexual assault [25].

Research in Western countries suggests that gender differences in the impact of SH also depend on its form. Women who have experienced gender harassment tend to develop more mental health problems than men with the same experience [11, 13, 16, 26]. Paradoxically, some studies showed a significant association between gender harassment and men’s mental health indicators [11, 13], while others did not [26]. Physical harassment has also yielded conflicting results regarding gendered mental health differences [11, 16]. Therefore, it is uncertain whether gender discrepancies exist regarding the associations of gender harassment and unwelcome sexual attention with the mental health of college students in the Chinese context.

### The Effect of Social Support

Social support is an umbrella term for any form of assistance received from another person [23]. It convinces people that they are cared for, loved, and are members of a network of mutual obligations [21–23]. Instrumental (material and financial support), emotional (psychological, emotional, and accepted support), and informational resources (advice and guidance) are key to social support [21, 23]. We measured financial, emotional, and learning support as their proxies. Researchers proposed two mechanisms to explain the health benefits of support: the main effect model and the stress-buffering hypothesis [21]. The former posits that regardless of the level of stress, social support can directly improve an individual’s well-being by ensuring personal esteem and satisfying the basic needs of companionship, intimacy, and a sense of belonging. The latter posits that social support can be protective against the adverse effects of stressful events in the short and long term by reducing an individual’s negative evaluation of stressful events, increasing their resources, and facilitating successful resolution or coping [21].

According to both hypotheses, when subjected to SH, college students embedded in support networks and with high social support will report fewer psychological problems than those without support. Though many empirical studies confirm the main effect [27, 28], only limited studies in Western countries examine the moderating effect of social support on the relationship between SH and mental health [2, 29, 30]. Some studies have suggested that not all types of support advocate the stress-buffering hypothesis [29, 30]. Furthermore, theoretical research also suggests that people develop expectations and preferences for specific types of support [23]. When support matches their needs or preferences, students are generally satisfied with and benefit from it. Supportive information that does not provide the preferred type of support may exacerbate or fail to address stress [31]. Many support-reactivity studies found emotional support more effective for psychological stress recovery [31, 32]. However, some researchers argued that instrumental support is better for coping with stress because it carries emotional meaning along with addressing material challenges [31, 33]. Therefore, it remains unclear whether all types of social support can reduce the risk of psychological problems arising from SH.

Additionally, theoretical research also posits that social support’s protective role depends on the stressful situation [31]; only when a person is under a high level of stress can support resources protect against adverse health effects [21, 23]. This suggests that the buffering role of each type of support varies depending on the form of SH. Unwelcome sexual attention exerts more psychological pressure than gender harassment [12]. Thus, social support may effectively buffer the effect of unwelcome sexual attention on students’ mental health. Meanwhile, the effectiveness of each type of support may also depend on gender [32, 34]. Nevertheless, existing studies have never directly tested these propositions.

### Problem Statement

Victims of SH often experience immediate mental health problems and chronic effects that can impact their adjustment for years [3–5]. SH is common in China [7, 35]. A survey of students at 6 universities in Beijing in 2019 reported that of 872 respondents, 59.52% experienced SH. Furthermore, the proportion of women who suffered SH was 63.03%, while that of men was 55.45% [36]. Though it is known that men suffer from SH too, few studies take an inclusive approach to investigating the relationship between SH and mental health among both women and men in China. Moreover, according to TTSC [8, 19, 20], there may be coping resources that can decrease the adverse effects of harassment. However, there is scant knowledge on how buffer resources and different types of support to reduce the impact of harassment on health are gendered. The current study contributes to previous research by investigating how—if at all—the relationship between SH and mental health is gendered and if social support protects the mental health of both genders when they are subjected to SH.

### Research Questions

Using a national sample from the Third Survey of Chinese Women’s Social Status, this study seeks to address: 1) Are gender harassment and unwelcome sexual attention related to the mental health of both genders in Chinese college students and, if so, do these relationships differ between genders? 2) Does financial, emotional, or learning support moderate the associations between the two forms of harassment and mental health among men and women?

## Methods

### Participants and Procedure

Statistics from China showed that in 2020, there were more than 30 million undergraduate students, more than 2 million master’s students, and 466,549 doctoral students. Female students accounted for 50.96%, 52.53%, and 41.87%, respectively [37]. We obtained data from a survey of college students from the Third Survey of Chinese Women’s Social Status, conducted by the All-China Women’s Federation (ACWF) and the National Bureau of Statistics between April and July 2011. The survey adopted a combination of quota and random sampling. First, according to the level of regional economic development, five destinations from cities with multiple universities were selected, namely Beijing, Nanjing, Wuhan, Xi’an, and Lanzhou. Thereafter, according to the university tiers, three national key- and non-key universities were selected from each city, for a total of 15 universities, including Peking University, Capital Normal University, Capital University of Economics and Business, etc. Finally, respondents were randomly selected from each university and classified by gender and academic degree. The student life adviser informed the respondents to go to a designated classroom but did not accompany them. Investigators were appointed by ACWF and did not know the respondents personally. They guaranteed the respondents’ anonymity and the right to withdraw at any time. After obtaining informed consent forms, the investigators administered questionnaires on the spot and collected them immediately after completion. The final sample comprised 5,032 participants. Sample characteristics are shown in Table 1.

**TABLE 1:** Sample characteristics (China, 2011).

	All	Missings
Characteristics	*n* = 5,032	%
Age (years): mean (SD)	23.35 (3.44)	0.18
Gender: n (%)		0.14
Female	2,531 (50.30%)	
Male	2,494 (49.56%)	
Academic degree: n (%)		0.12
Undergraduate	2,818 (56.00%)	
Master	1,543 (30.66%)	
PhD	665 (13.22%)	
University tiers: n (%)		0
Non-key university	1,978 (39.31%)	
Key university	3,054 (60.69%)	
Region: n (%)		0
Beijing	923 (18.35%)	
Nanjing	920 (18.29%)	
Wuhan	880 (17.49%)	
Xi’an	891 (17.71%)	
Lanzhou	1,417 (28.17%)	
Father’s education level: n (%)		0.79
Junior high school and below	1,915 (38.06%)	
High school	1,828 (36.33%)	
Junior college and above	1,249 (24.82%)	

### Measures

#### Mental Health

The self-assessment scale was compiled by the expert group of ACWF, with eight questions, namely ‘Have you experienced the following situations in the past month: 1) Being unable to fall asleep (insomnia), 2) Feeling physically and mentally exhausted, 3) Feeling irritable and easily angered, 4) Crying easily or wanting to cry, 5) Not [being] interested in anything, 6) Feeling lonely, 7) Feeling useless, and 8) Feeling that life is boring’. Responses were recorded on a four-point scale (0 = never; 3 = frequently). Other than the feeling of loneliness, the remaining seven questions came from the Chinese version of the Zung Self-rating Depression Scale (SDS) [38]. SDS is a widely used instrument and is often validly and reliably used to measure the mental health of Chinese students [39, 40]. In this study, Cronbach’s alpha was 0.83, indicating the scale has good reliability. After reversing the score of all items; the total score, obtained by summing the eight items (Mean = 17.00, SD = 4.60), was used as the dependent variable. A higher score indicated fewer negative symptoms and better mental health.

#### Sexual Harassment

The participants indicated whether they had the following experiences: “showing you pornographic images or pictures you are unwilling to see,” “telling you sexually explicit jokes that you are unwilling to hear,” “committing physical behaviours on you that you are unwilling to accept,” and “asking you for sex that you are unwilling to accept.” They were classified into two forms, gender harassment and unwelcome sexual attention, as measured by combining the first and latter two experiences. Each form comprised a binary variable, coded as 1 if participants experienced one or two types of harassment and 0 otherwise.

#### Social Support

Financial, emotional, and learning support were measured through three questions: “When you encounter ‘financial difficulties’, ‘emotional difficulties’, or ‘learning difficulties’, does anyone provide you with advice or help?” (“no one,” “one or two people,” “three or more people,” or “no difficulties”).

#### Socio-Demographic Covariates

Personal characteristics included gender and academic degree (“undergraduate,” “master’s student,” “PhD student”). University tiers were measured as “non-key university” and “key university” by assessing the school’s status. The key university has more standardised and stricter systems that could deter SH [41]. Discipline was measured by merging the 12 majors into two categories to represent male- and female-dominated environments: science and engineering (science, engineering, agriculture, medicine, military science) and humanities and social sciences (philosophy, economics, law, education, literature, history, and management).

Family characteristics included father’s and mother’s education level, measured as “junior high school and below,” “high school,” and “junior college and above.” Family economic status was measured by asking: “Compared to your classmates, how does your family’s economic condition?” (1 = very bad; 5 = very good).

### Data Analysis

Given that the overall mental health score was modelled as a continuous variable, we employed the ordinary least squares (OLS) regression. We developed three models to examine the association between SH and mental health: Model 1 only included gender harassment and unwelcome sexual attention as independent variables; Model 2 added socio-demographic covariates to enhance the internal validity of the associations; Model 3 further included the interactions between two forms of harassment and gender to examine whether the associations differed by gender.

Following Cohen’s suggestion, we adopted moderator analysis to determine whether social support acts as a buffer [42]. Using the female samples, we created three groups of models. Based on Model 2, Models 4A–6A incorporated financial, emotional, and learning support, respectively, to examine their main effects. Models 4B–6B further added the interactions between SH and the three types of support, respectively, to test the buffering effects of social support. Similarly, using the male samples, we created three groups of models to test the main and buffering effects of social support on men’s mental health (Models 7A–9A and 7B–9B).

## Results

### Gender Differences in the Relationship Between Sexual Harassment and Mental Health

In Table 2, Model 1 indicates that both gender harassment and unwelcome sexual attention were significantly negatively correlated with mental health. The standardised regression coefficient of the latter was significantly larger than that of the former. That is, compared with students who did not experience gender harassment or unwelcome sexual attention, the mental health of students who experienced these seemed to be worse, especially among those who experienced unwelcome sexual attention.

**TABLE 2:** Gender differences in the relationship between sexual harassment and mental health (China, 2011).

	Model 1	Model 2	Model 3
Gender harassment (Ref. No)			
Yes	−0.46** (−0.74, −0.18)	−0.66*** (−0.95, −0.38)	−0.53** (−0.92, −0.14)
Unwelcome sexual attention (Ref. No)			
Yes	−1.38*** (−1.71, −1.04)	−1.17*** (−1.51, −0.83)	−1.19*** (−1.71, −0.67)
Gender (Ref. Male)			
Female		−1.62*** (−1.87, −1.36)	−1.53*** (−1.85, −1.21)
Academic degree (Ref. Undergraduate)			
Master		0.19 (−0.10, 0.47)	0.19 (−0.10, 0.48)
PhD		0.28 (−0.12, 0.68)	0.28 (−0.12, 0.68)
University tiers (Ref. Non-key)			
Key university		0.34* (0.07, 0.61)	0.35* (0.08, 0.62)
Discipline (Ref. Science and engineering)			
Humanities and social sciences		−0.23+ (−0.49, 0.02)	−0.23+ (−0.49, 0.02)
Father’s education (Ref. Junior high school and below)			
High school		0.13 (−0.19, 0.44)	0.13 (−0.19, 0.44)
Junior college and above		−0.27 (−0.71, 0.18)	−0.26 (−0.70, 0.18)
Mother’s education (Ref. Junior high school and below)			
High school		0.12 (−0.19, 0.44)	0.13 (−0.19, 0.45)
Junior college and above		0.74** (0.24, 1.24)	0.74** (0.25, 1.24)
Family economic status		0.56*** (0.36, 0.75)	0.56*** (0.36, 0.75)
Gender harassment x Gender			−0.29 (−0.86, 0.27)
Unwelcome sexual attention x Gender			0.04 (−0.64, 0.73)
F	48.62***	26.31***	22.63***
Adj *R* ^2^	0.02	0.06	0.06
N	5,032	4,858	4,858

Data in Models 1–4 was presented as regression coefficients (95% CI).

^+^ *p* < 0.10, *p < 0.05, **p < 0.01, ***p < 0.001.

After adding socio-demographic variables, Model 2 shows that, compared to Model 1, the coefficient and significance of gender harassment on college students’ mental health increased, while the coefficient of unwelcome sexual attention decreased slightly and the significance remained unchanged. Model 3 included the interactions between two forms of harassment and gender. It shows that the regression coefficients of the two interaction terms were not significant.

### The Effects of Social Support

In Table 3, Models 4A–6A show that both gender harassment and unwelcome sexual attention were significantly negatively associated with women’s mental health, with the standardised regression coefficient of the latter being slightly larger than that of the former. Financial and emotional support were positively correlated with women’s mental health. Compared to women who received no financial or emotional support when encountering financial or emotional difficulties, those who received support from at least one person had higher mental health scores. Women who had no financial, emotional, or learning difficulties had much higher mental health scores than those who experienced that without supporters.

**TABLE 3:** The relationship between sexual harassment, social support, and mental health among female college students (China, 2011).

	Model 4A (Financial)	Model 4B (Financial)	Model 5A (Emotional)	Model 5B (Emotional)	Model 6A (Learning)	Model 6B (Learning)
Gender harassment (Ref. No)						
Yes	−0.82*** (−1.24, −0.39)	−0.69 (−3.37, 1.99)	−0.81*** (−1.24, −0.38)	−0.97 (−2.92, 0.99)	−0.82*** (−1.25, −0.39)	−2.28 (−5.67, 1.11)
Unwelcome sexual attention (Ref. No)						
Yes	−1.13*** (−1.59, −0.66)	−3.96** (−6.63, −1.28)	−1.09*** (−1.55, −0.62)	−2.62** (−4.60, −0.64)	−1.11*** (−1.58, −0.65)	0.05 (−3.34, 3.44)
Social support (Ref. No one)						
One or two	0.93+ (−0.14, 2.01)	0.25 (−1.05, 1.55)	1.15** (0.33, 1.97)	0.76 (−0.23, 1.75)	0.44 (−0.99, 1.86)	0.41 (−1.39, 2.21)
Three or more	1.28* (0.19, 2.37)	0.50 (−0.81, 1.82)	1.18** (0.36, 2.00)	0.70 (−0.28, 1.68)	1.02 (−0.39, 2.43)	0.90 (−0.89, 2.68)
No difficulties	1.79** (0.67, 2.90)	1.22+ (−0.13, 2.56)	2.54*** (1.59, 3.49)	2.30*** (1.18, 3.42)	2.35* (0.40, 4.29)	1.95 (−0.41, 4.31)
Sexual harassment x social support						
Gender harassment x One or two		−0.39 (−3.14, 2.37)		0.37 (−1.71, 2.44)		1.38 (−2.09, 4.84)
Gender harassment x Three or more		0.21 (−2.57, 2.98)		0.16 (−1.89, 2.20)		1.57 (−1.86, 5.00)
Gender harassment x No difficulties		−0.10 (−2.95, 2.76)		−0.45 (−2.85, 1.94)		0.25 (−5.62, 6.12)
Unwelcome sexual attention x One or two		3.24* (0.47, 6.01)		1.30 (−0.81, 3.40)		−1.32 (−4.79, 2.16)
Unwelcome sexual attention x Three or more		2.89* (0.10, 5.69)		1.94+ (−0.15, 4.03)		−1.16 (−4.60, 2.29)
Unwelcome sexual attention x No difficulties		2.34 (−0.53, 5.21)		1.40 (−1.14, 3.95)		0.92 (−3.89, 5.73)
F	8.72***	6.53***	9.86***	7.20***	8.58***	6.15***
Adj *R* ^2^	0.04	0.04	0.05	0.05	0.04	0.04
N	2,432	2,432	2,431	2,431	2,431	2,431

Data in Models 4A–6B was shown as regression coefficients (95% CI).

^+^ *p* < 0.10, *p < 0.05, **p < 0.01, ***p < 0.001.

All models involved socio-demographic covariates, namely gender, academic degree, university tiers, discipline, father’s and mother’s education level, and family economic status.

After adding the interactions, Models 4B–6B show that the interactions of unwelcome sexual attention with financial and emotional support were significant, and the regression coefficients were positive. Among women who received unwelcome sexual attention, those with at least one person providing financial support, and those with three or more people providing emotional support, had higher mental health scores than those without support networks.

In Table 4, Models 7A–9A show that gender harassment and unwelcome sexual attention were still significantly negatively correlated with men’s mental health, while the standardised regression coefficient of unwelcome sexual attention was greater than that of gender harassment. All three types of support were positively related to men’s mental health. Compared with men having no support network when encountering difficulties, those with over one person providing economic or learning support, and those with three or more people providing emotional support had much better mental health.

**TABLE 4:** The relationship between sexual harassment, social support, and mental health among male college students (China, 2011).

	Model 7A (Financial)	Model 7B (Financial)	Model 8A (Emotional)	Model 8B (Emotional)	Model 9A (Learning)	Model 9B (Learning)
Gender harassment (Ref. No)						
Yes	−0.54** (−0.91, -0.18)	−1.23 (−2.88, 0.43)	−0.55** (−0.92, −0.19)	−0.37 (−1.42, 0.67)	−0.53** (−0.90, −0.17)	1.65+ (−0.20, 3.50)
Unwelcome sexual attention (Ref. No)						
Yes	−1.21*** (−1.70, −0.71)	−2.92** (−5.11, −0.72)	−1.21*** (−1.71, −0.72)	−2.28** (−3.73, −0.83)	−1.22*** (−1.71, −0.72)	−3.12* (−5.81, −0.43)
Social support (Ref. No one)						
One or two	0.98* (0.22, 1.75)	0.32 (−0.66, 1.31)	0.45 (−0.10, 1.00)	0.34 (−0.38, 1.06)	0.90+ (−0.03, 1.83)	1.73* (0.42, 3.03)
Three or more	1.77*** (1.00, 2.54)	1.09* (0.11, 2.08)	0.51+ (−0.05, 1.07)	0.43 (−0.31, 1.17)	1.65*** (0.73, 2.57)	2.48*** (1.18, 3.78)
No difficulties	1.48** (0.61, 2.34)	1.35* (0.26, 2.44)	2.27*** (1.59, 2.96)	2.16*** (1.27, 3.06)	2.70*** (1.40, 3.99)	3.98*** (2.27, 5.69)
Sexual harassment x social support						
Gender harassment x One or two		0.95 (−0.80, 2.70)		−0.19 (−1.38, 1.01)		−2.27* (−4.21, −0.32)
Gender harassment x Three or more		0.93 (−0.82, 2.69)		−0.31 (−1.52, 0.90)		−2.28* (−4.20, −0.36)
Gender harassment x No difficulties		−0.74 (−2.71, 1.24)		0.14 (−1.37, 1.64)		−2.29 (−5.17, 0.58)
Unwelcome sexual attention x One or two		1.73 (−0.59, 4.06)		1.26 (−0.38, 2.90)		2.06 (−0.74, 4.85)
Unwelcome sexual attention x Three or more		1.89 (−0.44, 4.21)		1.39 (−0.28, 3.07)		2.09 (−0.69, 4.86)
Unwelcome sexual attention x No difficulties		1.80 (-0.83, 4.42)		0.48 (−1.55, 2.51)		−0.20 (−3.90, 3.50)
F	7.76***	6.19***	9.42***	6.77***	8.00***	6.09***
Adj *R* ^2^	0.04	0.04	0.05	0.05	0.04	0.04
N	2,424	2,424	2,425	2,425	2,424	2,424

Data in Models 7A–9B was shown as regression coefficients (95% CI).

^+^ *p* < 0.10, *p < 0.05, **p < 0.01, ***p < 0.001.

All models included socio-demographic variables, namely gender, academic degree, university tiers, discipline, father’s and mother’s education level, and family economic status.

After incorporating the interactions, Models 7B–9B show that the interaction between gender harassment and learning support was significant, and the regression coefficient was negative. That is, among men suffering from gender harassment, those with over one learning supporter had lower mental health scores than those without a supporter.

## Discussion

This study primarily aimed to examine the associations between different forms of SH and mental health among male and female college students in the Chinese context, and whether the associations differed by gender. Aligning with previous research, gender harassment is very common [43]. Men reported higher rates of gender harassment, whereas women faced more unwelcome sexual attention (see Supplementary Table). Both forms of harassment were negatively associated with mental health after controlling for the effect of gender and did not differ by gender. This suggests that the associations between the two forms of harassment and mental health in men and women are equally prominent. This finding is contrary to the general perception of Chinese people that SH does not endanger men [1, 18]. When faced with SH, which may lead to losing control over their personal or professional lives, men and women suffer the painful consequences equally. This outcome may be particularly true for college students, who are relatively sexually inexperienced and vulnerable to gossip and judgment from people around them [29, 44], and lack sufficient emotional control and coping skills.

Second, we aimed to investigate the moderating role of social support in the relationships between different forms of harassment and women’s and men’s mental health. We found that different types of support have different implications for the mental health harm caused by the two forms of harassment among both genders. Specifically, for female college students who have experienced harassment, financial and emotional support play an important role in improving their mental health. Having one and more financial supporters can protect women’s mental health directly and also helps them cope with the threats of unwelcome sexual attention to their mental health. One explanation is that financial support can enable women to become less dependent on friends and suitors when participating in social activities. This may directly increase their self-esteem and reduce negative emotions such as anxiety, tension, and embarrassment. Aligning with research findings, another explanation is that poverty is a risk factor for SH. Women are less exposed to dating-related harassment when they become more economically independent [2]. Financial support may also reduce women’s fears and increase courage and confidence in facing unwelcome sexual attention, promoting their psychological health. Moreover, having three or more emotional supporters can buffer the negative association between unwelcome sexual attention and mental health. The stress-buffering hypothesis is supported only by adequate emotional supporters, possibly because physical harassment and explicit sexual requests would continue to threaten their mental health and may make them develop negative emotions such as anxiety, panic, and depression [3–5]. If surrounded by supportive relationships, their negative emotions may be resolved without requiring a specific supporter. They may subsequently develop less severe emotional problems and have a higher sense of belonging, intimacy, and happiness [28].

Contrarily, for male college students, financial and large-scale emotional support assisted the main effect proposition but did not moderate the associations between harassment and mental health. As previous research suggested, compared with men, women consider social support more important in reducing the risk of stressors [45]. Another explanation is that financial problems, poor peer relationships, and social anxiety may make women more susceptible to SH [3, 46]. Paradoxically, learning support can protect men’s mental health directly but also indirectly harm it when faced with gender harassment. Learning support affects only men probably because they generally perform worse than women, receive lower grades and display poorer study skills [47]. As men are often reluctant to seek academic assistance due to the influence of internalized masculine norms [48], obtaining support whenever they are academically struggling is important for them, and may greatly improve their happiness [49]. As for why learning support is accompanied by negative effects, one reason may be that men are experiencing gender harassment while receiving learning support. Some reports suggest that gender harassment is highly common in schools, and classmates and friends rank first among the perpetrators of sexual violence or harassment of men [7]. Moreover, seeking learning support from peers may also aggravate psychological and academic pressure in male students who conform to hegemonic norms. This may negatively affect their self-esteem and create anxiety regarding reaching peers’ achievements [50], which may be superimposed on negative emotions caused by gender harassment.

### Limitations and Strengths

This study has some limitations. First, the measurement of SH omitted some information involving the commencement, duration, and frequency of harassment. This disabled the determination of whether it continued during the investigation. The questionnaire did not measure sexual coercion, the identities and gender of the perpetrators, or the locations where the harassment occurred. Research suggested that SH by offenders of different identities and gender might cause varying degrees of harm to students’ health [51]. Second, due to cross-sectional data limitations, we could not determine causal relationships between SH, social support, and mental health.

The strengths of this study include a national sample involving both genders, novel research questions, and distinctions between two forms of harassment and three functions of social support.

### Implications for Future Research and Practice

The link between SH and mental health among Chinese college students remains under question. First, further research is needed to explore the association of harassment with other well-being indicators, such as quality of life. Additionally, more studies are needed to determine whether psychological problems caused by SH affect future harassment encounters to form a vicious cycle. Research suggested that high levels of well-being negatively predicted SH [29]. Second, investigating whether social support protects students with poor psychological health from SH is necessary. Other buffer factors, such as perceptions of social support quality which are consistently related to mental health, should be explored further [21, 23, 28]. Third, future studies should include adolescents, as multiple studies in Western countries involved adolescent SH [2, 3, 29]. The SH experiences of the subjects may have occurred during adolescence. As Chinese parents consider “sex” a taboo, SH is rarely included in surveys and studies of adolescents. Fourth, longitudinal studies to reveal the link mechanism between SH, social support, and the mental health of college students are essential.

This study has important implications for practice, most importantly in creating a positive school environment with zero tolerance toward any form of SH. Student life counsellors and teachers must know that SH can be a significant trigger for students’ mental health problems. Clearly, women are not the only victims because men were equally psychologically harmed when facing SH. Interventions that provide students with necessary financial assistance and develop a range of activities that enhance their peer relationships can protect victims of SH from developing serious emotional problems. Additionally, family and school education should include sexuality to reduce the stigma and discrimination associated with SH, enabling them to report and seek help proactively, reducing psychological harm.

### Conclusion

This study is the first to explore the adverse psychological consequences of different forms of SH in both male and female Chinese college students. Inconsistent with the general perception, gender harassment and unwelcome sexual attention are negatively associated with students’ mental health, independent of gender. This study also prompts people to think about measures preventing students from developing mental health issues when they experience different forms of SH. In line with TTSC [8, 19, 20] and the stress-buffering hypothesis [21], financial and large-scale emotional support would help women recover and protect them from developing mental disorders caused by unwelcome sexual attention. However, neither of these two types of support are buffer factors for men. Contrary to the buffering hypothesis, psychological problems of men who experienced gender harassment increase after seeking learning support. This information may help administrators and teachers identify the causes of poor mental health and design targeted interventions to address the negative consequences of SH on college students.
