# Supplementary material for: The Association Between Sexual Harassment and Mental Health Among Chinese College Students: Do Gender and Social Support Matter?
Source: Int J Public Health. 2022 Sep 1;67:1604922. doi: 10.3389/ijph.2022.1604922 (PMC9474648; doi:10.3389/ijph.2022.1604922)
Supplement: Supplementary file 1 [file Table1.DOCX]

Supplemental Table

“The Association between Sexual Harassment and Mental Health among Chinese College Students: Do Gender and Social Support Matter?”

Table. Sexual harassment of college students with different personal and family characteristics (China, 2011)

|  | **Gender harassment** | *χ^2^* test | **Unwelcome sexual attention** | *χ^2^* test |
| --- | --- | --- | --- | --- |
| Total | 1,659 (32.97) |  | 945 (18.78) |  |
| Gender  Male  Female | 953 (38.21)  703 (27.78) | 61.92*** | 378 (15.16)  566 (22.36) | 42.76*** |
| Academic degree  Undergraduate  Master  PhD | 949 (33.68)  489 (31.69)  218 (32.78) | 1.79 | 523 (18.56)  303 (19.64)  117 (17.59) | 1.45 |
| University tiers  Non-key university  Key university | 706 (35.69)  953 (31.20) | 10.94** | 398 (20.12)  547 (17.91) | 3.85+ |
| Discipline  Science and engineering  Humanities and social sciences | 794 (33.00)  863 (32.96) | 0.001 | 400 (16.63)  544 (20.78) | 14.18*** |
| Father’s education level  Junior high school and below  High school  Junior college and above | 632 (33.00)  601 (32.88)  417 (33.39) | 0.09 | 311 (16.24)  368 (20.13)  259 (20.74) | 13.42** |
| Mother’s education level  Junior high school and below  High school  Junior college and above | 891 (33.26)  490 (32.19)  269 (33.92) | 0.83 | 455 (16.98)  330 (21.68)  154 (19.42) | 14.27** |
| Family economic status  Very bad  Bad  Fair  Good  Very good | 98 (38.74)  420 (34.97)  903 (31.02)  210 (37.97)  5 (35.71) | 17.16** | 43 (17.00)  212 (17.65)  535 (18.38)  135 (24.41)  4 (28.57) | 14.18** |

Data was presented as *n* (*%*).

+*p* < 0.10, ***p* < 0.01, ****p* < 0.001.
